# 11-*epi*-Sinulariolide Acetate Reduces Cell Migration and Invasion of Human Hepatocellular Carcinoma by Reducing the Activation of ERK1/2, p38MAPK and FAK/PI3K/AKT/mTOR Signaling Pathways

**DOI:** 10.3390/md12094783

**Published:** 2014-09-12

**Authors:** Jen-Jie Lin, Jui-Hsin Su, Chi-Chu Tsai, Yi-Jen Chen, Ming-Hui Liao, Yu-Jen Wu

**Affiliations:** 1 Graduate Institute of Veterinary Medicine, National Pingtung University of Science and Technology, Pingtung 91202, Taiwan; E-Mail: q87634@hotmail.com; 2 National Museum of Marine Biology and Aquarium, Pingtung 94450, Taiwan; E-Mail: x2219@nmmba.gov.tw; 3 Kaohsiung District Agricultural Improvement Station, Pingtung 900, Taiwan; E-Mail: tsaicc@mail.kdais.gov.tw; 4 Department of Physical Medicine and Rehabilitation, Kaohsiung Medical University Hospital, Kaohsiung 80761, Taiwan; E-Mail: chernkmu@gmail.com; 5 Department of Beauty Science, Meiho University, Pingtung 91202, Taiwan

**Keywords:** 11-*epi*-sinulariolide acetate, hepatocellular carcinoma, migration, invasion, matrix metalloproteinase, ERK1/2, p38MAPK

## Abstract

Cancer metastasis is one of the major causes of death in cancer. An active compound, 11-*epi*-sinulariolide acetate (11-*epi*-SA), isolated from the cultured soft coral *Sinularia flexibilis* has been examined for potential anti-cell migration and invasion effects on hepatocellular carcinoma cells (HCC). However, the molecular mechanism of anti-migration and invasion by 11-*epi*-SA on HCC, along with their corresponding effects, remain poorly understood. In this study, we investigated anti-migration and invasion effects and the underlying mechanism of 11-*epi*-SA in HA22T cells, and discovered by trans-well migration and invasion assays that 11-*epi*-SA provided a concentration-dependent inhibitory effect on the migration of human HCC HA22T cells. After treatment with 11-*epi*-SA for 24 h, there were suppressed protein levels of matrix metalloproteinase-2 (MMP-2), matrix metalloproteinase-9 (MMP-9) and urokinase-type plasminogen activator (uPA) in HA22T cells. Meanwhile, the expression of tissue inhibitor of metalloproteinase-1 (TIMP-1) and metalloproteinase-2 (TIMP-2) were increased in a concentration-dependent manner. Further investigation revealed that 11-*epi*-SA suppressed the phosphorylation of ERK1/2 and p38MAPK. The 11-*epi*-SA also suppressed the expression of the phosphorylation of FAK/PI3K/AKT/mTOR pathways.

## 1. Introduction

Hepatocellular carcinoma (HCC) is the second leading cause of cancer death in Taiwan due to its high recurrence and poor prognosis [1]. The high mortality rate is primarily related to tumor invasion and distant metastasis [2]. Previous studies have suggested that tumor cell motility and invasion are related to local and distant metastasis, and that various signaling molecules are involved in the metastatic process [3,4].

The change in adhesive capability between tumor cells and extracellular matrix (ECM) is one key factor in cancer cell metastasis. The ECM affects the biological behavior of cancer cells, including the regulation of cell attachment, tumor development, and metastasis [5]. The degradation of the ECM is mediated by proteases, such as serine proteinase and the matrix metalloproteinases (MMPs). Matrix metalloproteinase-2 (MMP-2) and matrix metalloproteinase-9 (MMP-9) are highly expressed in various malignant tumors and are involved in the degradation and breakdown of the environmental extracellular matrix (ECM) and the basement membrane, promoting cancer metastasis [6,7]. MMP-2 and MMP-9, activated by plasmin, are markers associated with the tumor invasion and metastasis [8]. Plasmin is derived from cleavage of the specific peptide bond in plasminogen, activated by various enzymes, of which urokinase plasminogen activator (uPA) is one key enzyme. Urokinase plasminogen activator receptor (uPAR) is a cofactor for plasminogen activation through uPA. Once plasmin is activated by uPA enzyme cascade, MMPs are activated, leading to the degradation of type IV collagen. Degradation of type IV collagen breakdowns the basement membrane, and leads to increased cell motility. uPA is also involved in the proliferation, migration, adhesion, and angiogenesis of tumors [9]. MMP-2, MMP-9 and uPA act as pivotal roles in degrading ECM and are involved in tumor metastasis and invasion [10]. Suppression of MMP-2, MMP-9 and uPA activity and expression could be a valid strategy to prevent tumor metastasis and invasion.

A number of pathological states, including cancer, inflammation, and vascular diseases are associated with increased proteinase inhibitors. Tissue inhibitors of metalloproteinase (TIMPs), the endogenous inhibitors of the endopeptidases of the matrix metalloproteinase families, act through the formation of a tight complex with their cognate enzymes and inhibit the activities of MMPs [11]. The imbalance between the TIMPs and MMPs may contribute to degradation or deposition of the ECM [12,13].

Mitogen-activated protein kinases (MAPKs) are serine/threonine protein kinases involved in different cellular responses, and regulate cell proliferation, differentiation and apoptosis. The MAPK signaling pathway is one important target in the development of anti-cancer therapies [14,15]. It has been reported that invasion and metastasis of HCC cells requires specific intracellular signaling cascade activations, among which the ERK1/2, p38MAPK and JNK signaling pathway is considered crucial [16,17,18,19]. An active compound, 11-*epi*-sinulariolide acetate (11-*epi*-SA), isolated from the cultured soft coral *Sinularia flexibilis*, has been shown to suppress inflammatory response and bone destruction in adjuvant induced arthritis and inhibits gene expression of cyclooxygenase-2 and interleukin-8 through attenuation of calcium signaling in EGF-stimulated epidermoid carcinoma cell [20,21]. The anti-metastatic effects of 11-*epi*-SA on HCC have yet to be evaluated. In the present study, we investigated the potential anti-metastatic effects of 11-*epi*-SA on hepatoma cell (HA22T) and the underlying mechanisms.

## 2. Results and Discussion

### 2.1. Effects of 11-epi-SA on HA22T Cells Viability

**Figure 1:**
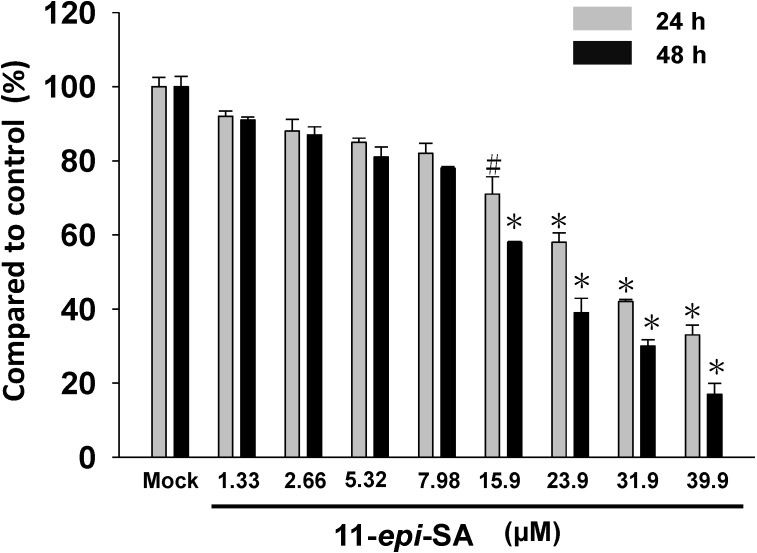
Evaluation of the cell viability effects of 11-*epi*-sinulariolide acetate (11-*epi*-SA) on HA22T cells. The cell viability of HA22T was concentration-dependently suppressed by MTT assay (* *p* < 0.001, ^#^ *p* < 0.05). Mock: the DMSO-treated cell. The experiments were repeated three times.

The results illustrate the anti-proliferation effects of 11-*epi*-SA at various concentrations (1.33~39.9 μM) on hepatocellular carcinoma cells (HA22T). The 11-*epi*-SA exhibited anti-proliferation activity against HA22T cells in a concentration-dependent manner (Figure 1). At a concentration of 15.9 μM, 11-*epi*-SA significantly inhibited the proliferation of HA22T cells, but at a concentration below 7.98 μM, the anti-proliferative effect was not obvious (Figure 1). We chose a concentration range of 11-*epi*-SA lower than 7.98 μM for all subsequent experiments. The concentrations of 11-*epi*-SA in this study have been examined in normal skin cell lines, HaCaT cells. The results (data not shown) exhibited that the cytotoxicity of 11-*epi*-SA is obviously lower in normal cells.

### 2.2. Inhibitory Effect of 11-epi-SA on Cell Migration and Invasion

**Figure 2:**
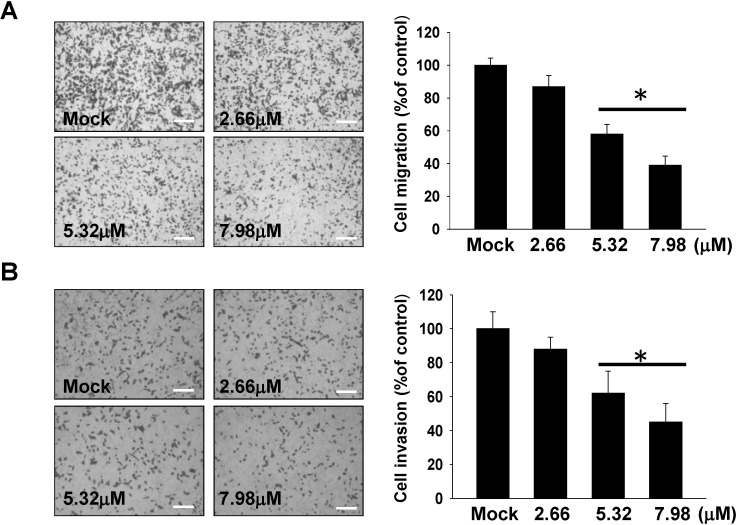
11-*epi*-SA inhibits HA22T cells migration and invasion. (**A**) 11-*epi*-SA inhibited transwell migration of HA22T cells; (**B**) 11-*epi*-SA inhibited invasion of HA22T cells. The transwell migration and matrigel invasion assay were performed as described in Materials and Methods. 11-*epi*-SA from 2.66 to 7.98 μM concentration-dependently decreased HA22T cells migration and invasion (* *p* < 0.001). Scale bar (**A**,**B**) = 20 μm; Mock: the DMSO-treated cell. The experiments were repeated three times.

Cell migration and invasion assays were performed to estimate the inhibitory effects of 11-*epi*-SA on the migratory characteristics of HA22T cells. Using a cell migration and invasion assay with a Boyden chamber, results showed that 11-*epi*-SA significantly reduced HA22T cells migration and invasion in a concentration-dependent manner. After treatment with 2.66, 5.32 and 7.98 μM of 11-*epi*-SA, the cell migration/invasion rates were 13%/12%, 41%/38%, and 60%/55%, respectively (Figure 2A,B) The results indicated increased inhibition of cell migration and invasion with increasing 11-*epi*-SA concentration.

### 2.3. Effects of 11-epi-SA on MMP-2, MMP-9, uPA, TIMP-1 and TIMP-2 Expression Levels

**Figure 3:**
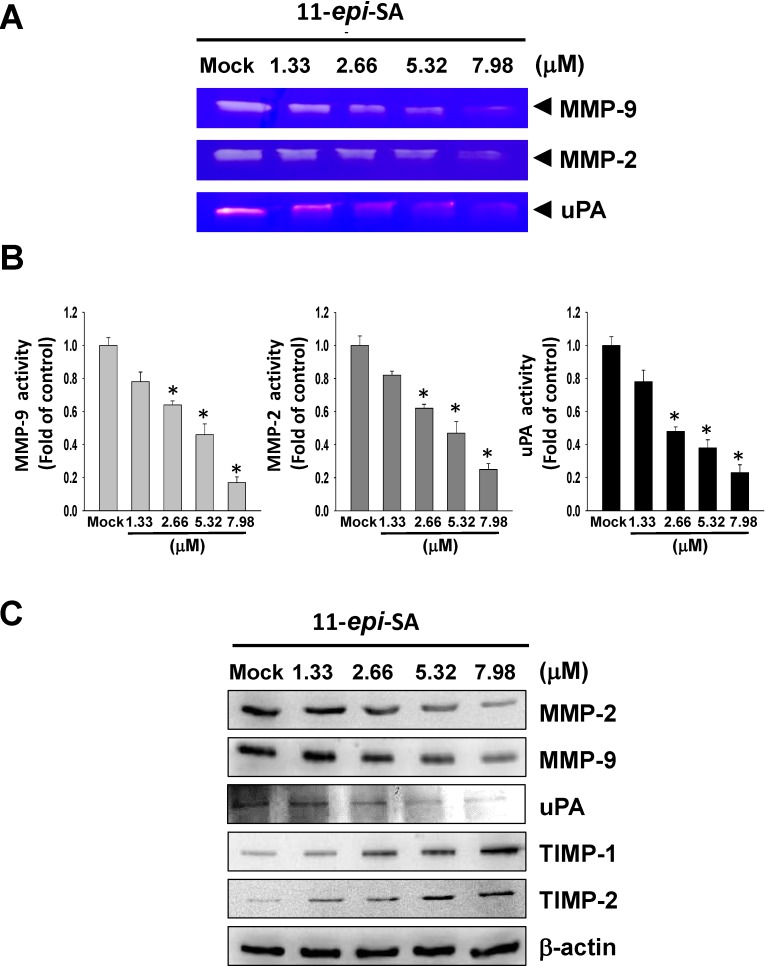
11-*epi*-SA suppressed MMP-2, MMP-9 and uPA activities and protein expression and increased TIMP-1/-2 protein expression. (**A**) The cells were treated with various concentrations of 11-*epi*-SA (1.33, 2.66, 5.32 and 7.98μM) for 24h.The conditioned media were collected and then MMP-2, MMP-9 and uPA activities were determined by gelatin zymography and casinezymography, respectively; (**B**) Quantification of MMP-2, MMP-9 and uPA; (**C**) The protein levels of MMP-2, MMP-9, uPA, TIMP-1 and TIMP-2 were verified in HA22T cells treated with 11-*epi*-SA (1.33, 2.66, 5.32 and 7.98 μM) for 24 h by western blot analysis. Mock: the DMSO-treated cell.β-actin was used as the internal control.

MMP-2 and MMP-9, extracellular membrane degrading enzymes, have been identified as playing important roles in cancer cell metastasis and invasion [10,12]. MMP-2 and MMP-9 activities act as pivotal roles in cell proliferation, cell migration, and angiogenesis. Gelatin zymography was used for analysis of MMP-2 and MMP-9 activities. As shown in Figure 3A,B, 11-*epi*-SA significantly reduced MMP-2 and MMP-9 activities in a concentration-dependent manner. MMPs are activated by proteolysis of pro-MMPs by uPA. Therefore, we next investigated the role of uPA in 11-*epi*-SA-mediated regulation of MMP-2 and MMP-9. Casein zymography revealed that 11-*Epi*-SA significantly decreased uPA activity (Figure 3A).

We used western blot analysis to investigate the inhibitory effect of 11-*epi*-SA on associated protein levels of migration and invasion. HA22T cells were treated with 11-*epi*-SA (1.33~7.98 μM) for 24 h and then subjected to western blot analysis. Figure 3B showed that 11-*epi*-SA significantly reduced the protein levels of MMP-2, MMP-9 and uPA, and increased TIMP-1 and TIMP-2 protein expression in a concentration-dependent manner.

### 2.4. The ERK1/2 and p38MAPK Signaling Pathways Are Involved in the Anti-Metastatic Mechanism of 11-epi-SA

**Figure 4:**
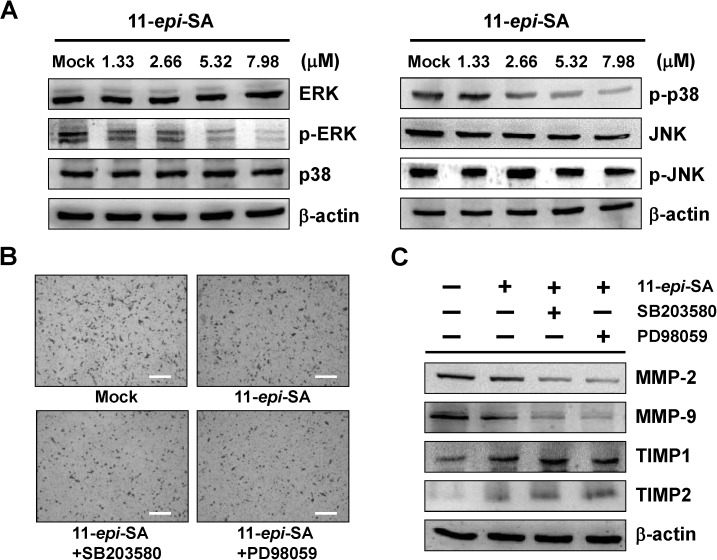
Effects of 11-*epi*-SA on MAPKs signaling pathway related proteins expression in HA22T cells. (**A**) The protein levels of ERK1/2, p-ERK1/2, p38MAPK, p-p38MAPK, JNK, and p-JNK after treatment with various concentrations of 11-*epi*-SA were analyzed by western blotting; (**B**) HA22T cells were pretreated with SB203580 and PD98059 for 1 h and then incubated in the absence or presence of 11-*epi*-SA (7.98 μM) for 24 h. Cell invasiveness was measured using matrigel invasion assay; (**C**) The protein levels of MMP-2, MMP-9, TIMP-1 and TIMP-2 were analyzed in treated HA22T cells by western blot analysis. Scale bar (**B**) = 20 μm. Mock: the DMSO-treated cell. β-actin was used as the internal control.

MAPKs are well-established pathways that modulate MMPs expression. In human HCC cells, activation of MAPKs signaling pathway is required for the invasion process. Thus, we investigated the effects of 11-*epi*-SA on MAPKs signaling pathways using western blot analysis. Figure 4A shows that 11-*epi*-SA significantly reduced the expression of phosphorylated ERK1/2 and p38MAPK in a concentration-dependent manner, but not JNK (Figure 4A). In order to evaluate whether the inhibitory effect of 11-*epi*-SA on cell migration, cell invasion and MMP-2 and MMP-9 expression were correlated with inhibition of the ERK1/2 and p38MAPK signaling pathway, HA22T cells were pretreated with ERK1/2 inhibitor (PD98059, 2 μM) and p38MAPK inhibitor (SB203580, 20 μM) for 1h and then incubated with 11-*epi*-SA (7.98 μM) for 24 h. The results showed that treatment of HA22T cells with 11-*epi*-SA and pretreated with PD98059 and SB203580 significantly inhibited cell invasion and reduced MMP-2 and MMP-9 proteins expression. Meanwhile, the expression of TIMP-1 and TIMP-2 were increased (Figure 4B,C).

### 2.5. Effects of 11-epi-SA on PI3K/AKT/mTOR Signaling Pathways

**Figure 5:**
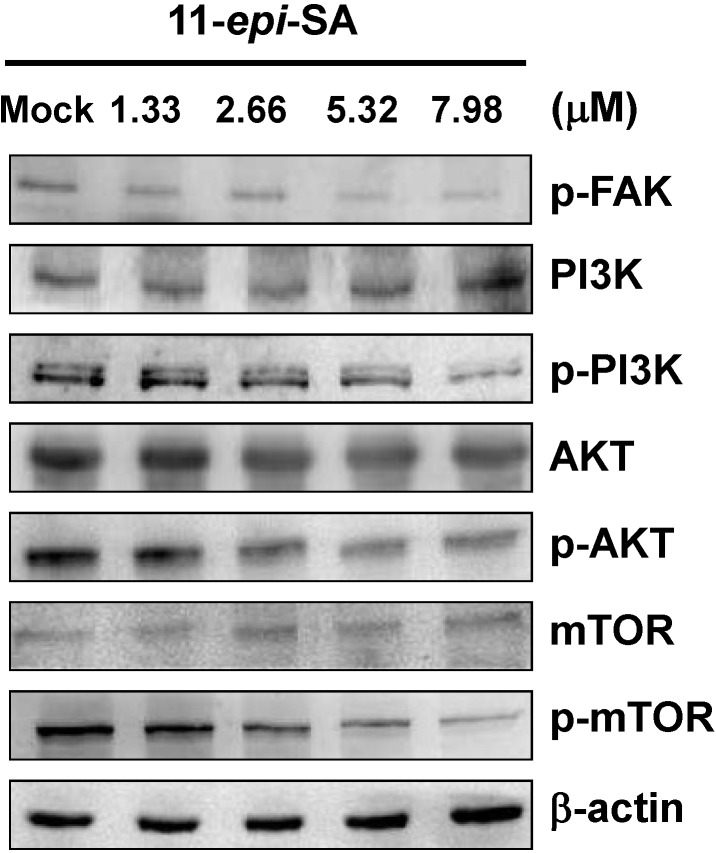
Effects of 11-*epi*-SA on FAK/PI3K/AKT/mTOR signaling pathways in HA22T cells. Western blotting data showed the changes of p-FAK, p-PI3K, PI3K, AKT, p-AKT, mTOR, and p-mTOR expression in HA22T cells treated with different concentrations of 11-*epi*-SA. Mock: the DMSO-treated cell. β-actin was used as the internal control.

We investigated the effects of 11-*epi*-SA on FAK/PI3K/AKT/mTOR pathways using western blot analysis. Results showed that 11-*epi*-SA decreased the phosphorylation of FAK, PI3K, AKT and mTOR in a concentration-dependent manner. The protein expressions of PI3K, AKT and mTOR were not changed after 11-*epi*-SA treatment (Figure 5).

### 2.6. Effects of 11-Epi-SA on Cell Migration and Invasion-Associated Protein Levels

**Figure 6:**
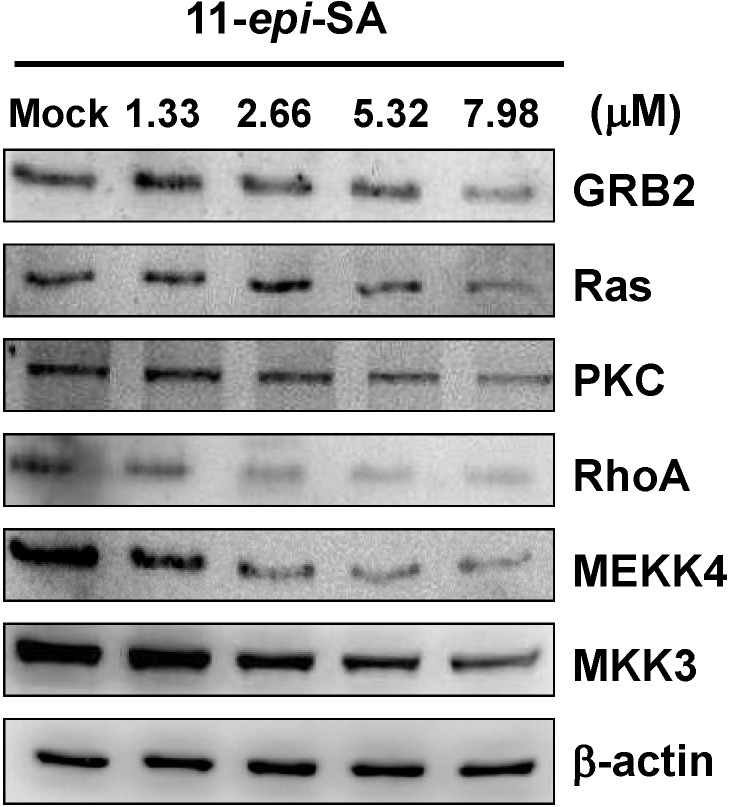
11-*epi*-SA inhibited the levels of associated protein in cell migration and invasion of HA22T cells. The levels of PKC, GRB2, Ras, Rho A, MEKK4 and MKK3 expression in HA22T cells treated with different concentrations of 11-*epi*-SA were estimated by western blot analysis. Mock: the DMSO-treated cell. β-actin was used as the internal control.

The levels of migration and invasion-associated proteins in HA22T after 11-*epi*-SA treatment were determined by western blot analysis. The results indicated that the levels of PKC, Ras, Rho A, growth factor receptor-bound protein 2 (GRB2), MEKK4 and MKK3 were lower than the control group after 11-*epi*-SA treatment (Figure 6).

### 2.7. Discussion

Cancer cell metastasis is related primarily to cell migratory ability and invasiveness. Cell migration and invasion mechanisms are related to binding of related growth factors to cell surface receptors, and the activation of downstream signaling pathways, leading to cytoskeletal reorganization and increased cell motility [22]. The development of anti-cancer therapies has been strongly targeted on these cancer migration and invasion related molecules for the control of metastatic diseases [23].

The anti-cancer effects of specific compounds from soft coral on different cancer cell lines have been identified [24,25,26,27,28,29,30]. Of many anti-cancer therapeutic targets, anti-metastatic effect of the specific anti-cancer agent is one important concern in the prediction of treatment response. 11-*epi*-SA is an active compound extracted from the cultured soft coral *Sinularia flexibilis*. The exact anti-migration and anti-invasion molecular mechanism of 11-*epi*-SA has not yet been reported. In the present study, to elucidate the effect of 11-*epi*-SA on cell migration and invasion, Boyden chamber migration and invasion assay were carried out. The results indicated that 11-*epi*-SA inhibited the migration and invasion of HA22T cells in a concentration-dependent manner (Figure 2). Our study also showed that in HA22T cells treated with 11-*epi*-SA at non-toxic doses (no more than 7.98 μM), cell migration and invasion were inhibited. These results implied that the inhibition of migration and invasion by 11-*epi*-SA in HA22T cells was not due to cytotoxicity.

It is well known that cancer cell metastasis involves multiple processes and various physiological changes. The MMPs-mediated degradation of the ECM is a critical step in tumor invasion and metastasis [8,31]. MMPs, especially MMP-2 and MMP-9, have been reported to act as important roles in tumor invasion and metastasis [10,12]. Activation of plasmin is mediated by various enzymes, and uPA activity is suggested to be the most sensitive predictor of HCC invasion and tumor recurrence [32]. Thus, secretion of MMP-2, MMP-9 and uPA by invasive carcinoma is thought to be important in promotion of tumor metastasis and invasion, primarily related to the degradation of ECM by these enzymes [33].

The inhibition of the activity of MMPs may be a useful strategy for controlling metastasis of cancer cells even in the early tumor stages [34]. TIMPs are inhibitors of endopeptidases of the matrix metalloproteinase families, and inhibit MMP activities to prevent extensive ECM degradation. The imbalance between TIMPs and MMPs activities may contribute to tumor metastasis and invasion. TIMP-1/-2 has been shown to have a statistically significant association with antimetastasis on HCC [35,36]. Chen *et al.* showed that dihydroaustrasulfone alcohol, isolated from marine coral, substantially suppressed the proliferation and invasiveness of a non-small cell lung carcinoma cell line (A594), with a notable decrease in MMP-2 and MMP-9 expression [37]. In the present study, we found that 11-*epi*-SA reduced the protein levels and activity of MMP-2, MMP-9 and uPA and simultaneously increased TIMP-1 and TIMP-2 protein levels (Figure 3). These results indicate that the inhibition of the migratory and invasive effect of 11-*epi*-SA on HA22T cells is correlated with the modulation of MMPs and their inhibitors.

Mitogen-activated protein kinases (MAPKs) include extracellular signal-regulated kinase (ERK1/2), p38MAPK and c-jun-N-terminal kinase (JNK1/2) [38]. MAPKs activation is followed by phosphorylation of various cytosolic proteins associated with cell migration, cell invasion, cell proliferation, cell differentiation, and cell apoptosis [39]. Recent reports indicate that MAPKs signaling pathways are involved in the regulation of MMPs and uPA expression in tumor cell invasion [40,41]. Reports also suggest that invasion and metastasis of HCC cells requires MAPKs signaling cascade activations [18,19]. In this study, we verified that 11-*epi*-SA has an inhibitory effect on migration and invasion through the suppression of MMP-2, MMP-9 and uPA on HA22T cells. Our data showed that 11-*epi*-SA suppressed the expression of phosphorylation of ERK1/2 and p38MAPK in a concentration-dependent manner, but not JNK1/2. The 11-*epi*-SA combined with ERK1/2 inhibitor (PD98059) and p38MAPK inhibitor (SB203580) significantly reduced HCC cell invasion and was accompanied by downregulation of MMP-2 and MMP-9 and upregulation of TIMP-1 and TIMP-2 (Figure 4).

Focal adhesion kinase (FAK) acts as an important protein in cell-ECM interactions that affect cell proliferation, migration and metastasis [42,43]. Phosphorylated FAK acting as a scaffold, regulates mainly focal adhesion signaling related to cell adhesion to ECM and MMPs-mediated matrix degradation [44]. The FAK/PI3K/AKT/mTOR signal transduction pathway is involved in cell proliferation, differentiation, survival, and migration [45]. Several studies have indicated that FAK/PI3K/AKT/mTOR signal pathway is involved in the regulation of MMP-2 and MMP-9 activity [46,47,48]. In our study, western blot results showed that 11-*epi*-SA decreased the phosphorylation of FAK, PI3K, AKT and mTOR. The protein expression of PI3K, AKT and mTOR were not changed after 11-*epi*-SA treatment (Figure 5).

GRB2 is an adaptor protein that binds to Son of Sevenless (SOS), encoding Ras-specific guanine nucleotide exchange factor (GEF), in the MAPK pathway. Binding of SOS to GRB2 in the plasma membrane sequentially activates Ras, Raf, MEK1/2, and ERK1/2 [49]. In invasive breast cancer cells, GBR2 is suggested to regulate the activation of the GTPases ARF1 and ARF6 [50]. The Rho family of GTPases regulates intracellular actin dynamics, cell spreading and migration, and the activity of rho proteins changes with the change in upstream GEF signal [51,52]. Some studies indicate inhibition of the RhoA reduced cancer cell migration and invasion [53,54]. In our study, western blot results showed that 11-*epi*-SA decreased the levels of GRB2, Ras, RhoA, MEKK4 and MKK3, which are also involved in cell migration (Figure 6).

**Figure 7:**
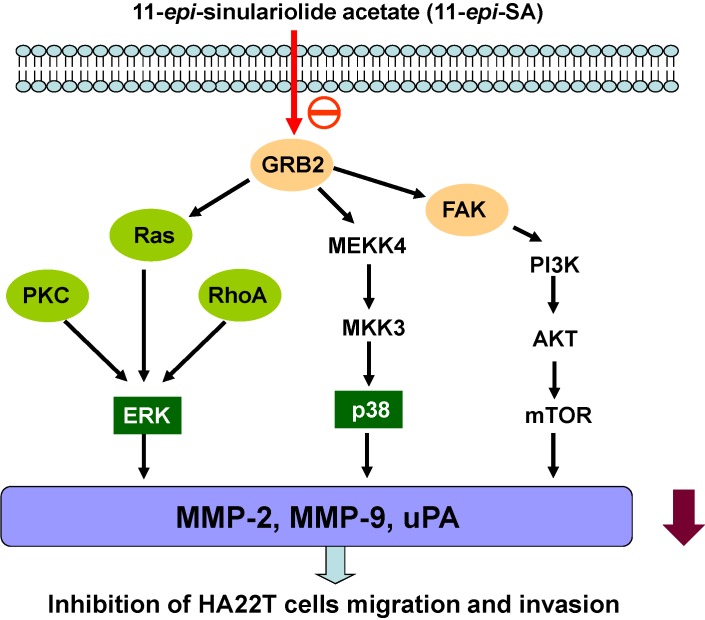
Molecular signaling pathways of the inhibitory effect of 11-*epi*-SA on hepatocellular carcinoma HA22T cell migration and invasion.

Based on our results, the proposed signaling pathways of the inhibitory effect of 11-*epi*-SA on HA22T cells migration and invasion are shown in Figure 7. 11-*epi*-SA was downregulated MMP-2, MMP-9 and uPA protein expression and inhibited metastatic effect through suppressed ERK1/2, p38MAPK and FAK/PI3K/AKT/mTOR signals on HA22T cells.

## 3. Material and Methods

### 3.1. Materials and Antibodies

11-*epi*-sinulariolide acetate (11-*epi*-SA) was extracted from cultured soft coral *Sinularia flexibilis*, following the protocol described by Hsieh *et al.* [55], and dissolved in DMSO. 3-(4,5-dimethylthiazol-2-yl)-2,5-diphenyltetrazolium bromide (MTT), Dimethyl sulfoxide (DMSO), Coomassie Blue R-250, Ponceau S, NaCl, NaN_3_, ZnCl_2_, CaCl_2_, Triton-X 100 and rabbit anti-human β-actin antibodies were obtained from Sigma (St Louis, MO, USA). Goat anti-rabbit and horseradish peroxidase conjugated IgG and PVDF (polyvinylidenedifluoride) membranes were obtained from Millipore (Bellerica, MA, USA). Chemiliminescent HRP substrate was purchased from Pierce (Rockford, IL, USA). Rabbit anti-humanprotein kinase C (PKC), mitogen-activated protein kinase kinase3 (MKK3), MAPK kinase kinasekinase4 (MEKK4), Focal adhesion kinase (FAK), growth factor receptor-bound protein 2 (GRB2), Rho A, mTOR and p-mTOR antibodies were obtained from Epitomics (Burlingame, CA, USA). Rabbit anti-human TIMP-1, TIMP-2, AKT and p-AKT antibodies were obtained from ProteinTech Group (Chicago, IL, USA). Rabbit anti-human matrix metalloproteinase-2 (MMP-2), matrix metalloproteinase-9 (MMP-9), uPA, c-jun *N*-terminal kinase (JNK), p-JNK, extracellular signal regulated kinases (ERK), phosphorylated extracellular signal regulated kinases (p-ERK), phosphoinositide 3-kinases (PI3K), phosphorylated-phosphoinositide 3-kinases (p-PI3K), c-jun, p-c-jun, p38 and p-p38antibodies were obtained from Cell Signaling Technology (Danvers, MA, USA).

### 3.2. Cell Culture and MTT Assay

Human hepatoma HA22T cells were purchased from Food Industry Research and Development Institute (Hsinchu, Taiwan). Cells were cultured as described in our previous report [30]. HA22T cells were treated with various concentrations of 11-*epi*-SA (0, 1.33, 2.66, 5.32, 7.98, 15.9, 23.9, 31.9 and 39.9 μM) and harvested after incubation for 24 h. The cell viability of HA22T cells after 11-*epi*-SA treatment was examined by MTT assay as described in our previous study [20]. HA22T cells were seeded on 24-well culture plates at a density of 1 × 10^5^ cells/well. After various concentrations of 11-*epi*-SA incubation for 24 h, 50 μL of MTT solution (1 mg/mL in PBS) was added to each well. Cells treated with DMSO were used as blank control. The cell culture plates were incubated at 37 °C for 4 h and then cells were lysed with 200 μL DMSO. The optimal density (OD) was measured at 595 nm by a microtiter ELISA reader (Bio-Rad, Hercules, CA, USA). All the experiments were repeated three times.

### 3.3. Cell Migration and Invasion Assay

The cell migration assay was performed according to the methods described by Neoh *et al.* [56]. HA22T cells were seeded into a Boyden chamber (Neuro Probe, Cabin John, MD, USA) at 10^4^ cells/well in serum-free media. HA22T cells with 11-*epi*-SA treatment (0, 2.66, 5.32 and 7.98 μM) were kept at 37 °C for 24 h to allow cell migration. For invasion assay, 10 μL Matrigel (25 mg/50 mL; BD Biosciences, MA, USA) was coated onto 8 μm pore-size polycarbonate membrane filters, and HA22T cells were plated in the upper chamber of the Matrigel-coated Transwell insert. The bottom chamber contained cell culture medium previously described by Yeh *et al.* [57]. The migrated and invaded cells on the lower chamber were fixed with 100% methanol and stained with 5% Giemsa (Merck, Germany). Cell numbers were counted using a 100× light microscope.

### 3.4. Determination of MMP-2/MMP-9 and uPA Activities by Gelatin Zymography

Gelatin and casein zymography assays were used to measure the activities of MMP-2, MMP-9 and uPA in conditional medium as previously described [58]. HA22T cells were treated with various concentrations of 11-*epi*-SA (1.33, 2.66, 5.32 and 7.98 μM) for 24 h. To analyze the secretion of MMP-2/-9 and uPA in culture media, the collected culture media were concentrated by a speed vacuum. The samples were separated by 10% SDS-PAGE containing 0.2% gelatin under non reducing conditions for MMP-2/-9 activity assay and containing 0.2% casein and 10 μg/mL plasminogen for uPA activity assay. The gels were washed in wash buffer (100 mM NaCl and 2.5% Triton X-100 in 50 mM Tris-HCl, pH7.5) three times. Then the gels were incubated in reaction buffer (200 mM NaCl, 0.02% NaN_3_, 1 μM ZnCl_2_, 1 mM CaCl_2_, 2% Triton-X 100, in 50 mM Tris-HCl, pH 7.5) at 37 °C for 24 h. The gels were stained with Coomassie Blue R-250, and destained and quantified using Image J 1.47 software (National Institutes of Health, Bethesda, MD, USA).

### 3.5. Western Blot Assay

The treated samples and the control samples (25 μg) were separated by 12.5% SDS-PAGE, and then transferred onto PVDF membrane for 1.5 h at 400 mA using Transphor TE 62 (Hoeffer) and then protein transfer was checked by staining with Ponceau S solution. The membranes were subsequently incubated with 5% dehydrated skimmed milk in PBS Buffer (10 mM NaH_2_PO_4_, 130 mM NaCl) to block nonspecific protein bindings, and then incubated with primary antibodies at 4 °C overnight. The primary anti-human MMP-2, MMP-9, uPA, TIMP-1, TIMP-2, GRB2, p-FAK, MEKK4, MKK3, Rho A, JNK, p-JNK, PKC, ERK, p-ERK, p38MAPK, p-p38MAPK, PI3K, p-PI3K, AKT, p-AKT, mTOR, p-mTOR and β-actin antibodies were used. The second antibodies (horseradish peroxidase conjugate goat anti-rabbit, 1:5000 in blocking solution) were added and incubated for 2 h at 4 °C and then visualized using chemiluminesence (Pierce Biotechnology, Rockford, IL, USA).

### 3.6. Statistical Analysis

Data analyses of MTT assays, cell migration and invasion assays were derived from three independent experiments. Tukey-Kramer test was used for data acquisition and analysis of variance (ANOVA), using Graphpad Instat 3 software (San Diego, CA, USA).

## 4. Conclusions

Overall, our results demonstrated for the first time that 11-*epi*-SA effectively inhibited migration and invasion of human HA22T cells through ERK1/2, p38MAPK and FAK/PI3K/AKT/mTOR signaling pathways, causing downregulation of MMP-2, MMP-9 and uPA expression as summarized in Figure 7. Based on these observations, we suggest that 11-*epi-*SA could be a potential candidate for development of preventive agents against hepatocellular carcinoma metastasis and invasion in the future.
